# Evidence-based policies in public health to address COVID-19 vaccine hesitancy

**DOI:** 10.2217/fvl-2022-0028

**Published:** 2023-04-04

**Authors:** Francesco Chirico, Jaime A Teixeira da Silva

**Affiliations:** ^1^Post-graduate School of Occupational Medicine, Università Cattolica del Sacro Cuore, Rome, Italy; ^2^Independent researcher, Ikenobe 3011-2, Kagawa-ken, 761-0799, Japan

**Keywords:** COVID-19, decision-making, healthcare professionals, infodemic, misinformation, public health policies, SARS-CoV-2, transparent communication, vaccines

## Abstract

A fundamental basis for effective health-related policymaking of any democratic nation should be open and transparent communication between a government and its citizens, including scientists and healthcare professionals, to foster a climate of trust, especially during the ongoing COVID-19 mass vaccination campaign. Since misinformation is a leading cause of vaccine hesitancy, open data sharing through an evidence-based approach may render the communication of health strategies developed by policymakers with the public more effective, allowing misinformation and claims that are not backed by scientific evidence to be tackled. In this narrative review, we debate possible causes of COVID-19 vaccine hesitancy and links to the COVID-19 misinformation epidemic. We also put forward plausible solutions as recommended in the literature.

## Sources of COVID-19 vaccine hesitancy & the importance of effective messaging

Vaccine hesitancy, defined as a *“delay in acceptance or refusal of vaccination despite [the] availability of vaccination services”* (p. 4163), is context-specific and varies, depending on many factors [1]. Inadequate communication by public health stakeholders can lower vaccine acceptance and contribute to vaccine hesitancy induced by false beliefs or attitudes about health and prevention, as well as low levels of knowledge and awareness of the recipients [1]. Vaccine hesitancy may even arise from a lack of trust in health authorities, physicians, or vaccines, the lack of clarity about vaccines' safety and efficacy, insufficient information, or misinformation. COVID-19 vaccine hesitancy needs to be addressed because the mass vaccination is ongoing in a bid to control the pandemic. To achieve this, the effectiveness and safety of vaccines must be proved [2].

COVID-19 vaccines are effective instruments against severe disease, hospitalization, and deaths because they provide recipients with a layer of immunity to combat infection, so they are considered the most effective preventive measure to reduce the burden and pressure created by this pandemic on healthcare systems [2]. An ambitious aim is to inhibit or block the emergence of new SARS-CoV-2 variants with the potential to escape the immune response provided by vaccines [2]. However, to ability to achieve population immunity by continued vaccinations to reduce SARS-CoV-2 circulation has been criticized because it will likely depend on the virus' endemic presence [3] and because immunity wanes over time [3]. COVID-19 vaccine booster doses should thus balance waning immunity and the evolution of new viral strains while prioritizing vulnerable and immunocompromised populations [4]. There is debate as to whether new strains of the Omicron variant of SARS-CoV-2 are able to evade immunity [5], thereby resisting existing COVID-19 vaccines [5]. Particularly for adults 60 years of age or older, there is debate about the magnitude of effectiveness of four doses of the BNT162b2 (Pfizer-BioNTech) messenger RNA (mRNA) vaccine compared with that conferred by three doses [6]. However, in some countries such as Israel, a fourth dose was approved for immunocompromised groups, and a fourth dose was offered to healthcare workers (HCWs) and individuals over 60 years [7].

Effective strategic communication is a prerequisite for building public trust in vaccines [8]. Vaccine hesitancy, especially among young parents who are concerned for their children, emerges from their lack of knowledge about potentially harmful adjuvants, preservatives, inactivating agents, or manufacturing residuals that exist in vaccines, and their fear that vaccines might cause autism, diabetes, or other diseases or disorders [8]. Mistrust in COVID-19 vaccines, i.e., vaccine hesitancy, emerged from misinformation about the virus, including misconstrued or unfounded beliefs associated with the origin of COVID-19 [9]. Vaccine hesitancy also arises from the existence of economic barriers [10] or due to the absence of ethical expertise in vaccine-related policy-making [11].

The spread of anti-vaccine views with different narratives, such as safety concerns, opposition to mask-wearing, and fears of restricted freedoms (e.g., of movement), propagated via the internet and social media, allowed conspiracy theories to develop [12]. Such conspiracy theories involve the use of emotive language, using powerful social media platforms such as Twitter to fuel their philosophies [13]. A social media-savvy community of engaged influencers that rely on scientifically-grounded insight into vaccines is necessary [13]. Vaccine hesitancy might also be related to philosophical or moral beliefs regarding ‘natural’ versus ‘artificial’ health and immunity [14].

The overarching message of previous paragraphs suggests that the message by health authorities has not been sufficiently convincing or effective to relay concerns. Given that the public is receptive to messaging, especially if health agencies make public announcements [12], policymakers should heed the public's concerns when considering their approaches to communication and vaccination education, including the ability to address negative sentiments and messaging, such as fear, risk, and anxiety, while taking positive actions, such as altruism [12]. Healthcare workers (HCWs) also need to be motivated to fortify their self-confidence and to dampen patients' defensiveness while providing accurate information that ensures that patients understand that vaccination efforts can reduce the risk of disease and death [15]. COVID-19 vaccine programs should thus provide a focused and empathetic response, while realistically noting risks, to counter distrust and misinformation [9].

In this section, we argued that messaging about the effectiveness of vaccines is essential for combating vaccine hesitancy.

## Constantly updated policies & public education to combat vaccine hesitancy

Vaccine acceptance results from multifactorial and complex decisions that depend on contextual factors, including communication, culture, politics, and barriers to accessibility, as well as individual and group differences, such as beliefs and attitudes toward public health interventions, trust in the government and pharmaceutical industries, awareness of the risk/benefit ratio, and characteristics of the vaccine, including adverse effects, vaccine schedule, and others [1,12]. According to the 3Cs model by the WHO, an individual's propensity to be vaccine-hesitant depends on three factors: confidence (i.e., trust in vaccine effectiveness and safety), complacency (or the perception of risk of the disease) and convenience (i.e., accessibility) [16]. These factors need to be addressed by local and central governments via updated policies for educating the public [16].

The WHO invited all countries to develop a COVID-19 vaccine safety communication plan, with the aim of more effective communication with the public, and to ultimately vaccinate as many people as possible. Essential to this plan is the establishment of strategic partnerships with key stakeholders, including HCWs, as trusted influencers and vaccinators, governmental and private bodies, as well as organizations, using open and transparent communication, based on evidence-based messaging [17]. To achieve this, it is essential to collect data on the safety and effectiveness of COVID-19 vaccines and analyze them through risk-benefit analyses, especially for population groups such as individuals who are medically vulnerable (i.e., with preconditions), workers with a high risk of getting infected (e.g., HCWs, non-healthcare frontline workers, essential workers, etc.), children, and pregnant women, before communicating the need to get vaccinated to the public. Any evidence-based policy in the public health field requires open data sharing between scientists, government health officials, HCWs, and policymakers [17]. A society's trust in vaccines is thus built on effective communication that highlights the benefits, realistically shows the risks, but also provides clear evidence about how such risks can be mitigated, or not [17]. For instance, a cost-benefit analysis suggested a higher risk of myocarditis in adults infected with SARS-CoV-2 than in those vaccinated with mRNA vaccines (BNT162b2 and mRNA-127), but a lower risk of myocarditis in younger people [18]. However, that study was not conclusive and additional clinical research is needed to verify its claims. Thus, cost-effectiveness analyses should be tailored to the needs of target populations. An evidence-based medicine (EBM) approach to vaccines thus requires the assessment of risk [19].

The European Medicines Agency (EMA) in the European Union (EU) and the Federal Drug Administration (FDA) in the US have authorized the use of vaccines that are currently being used in the campaign against COVID-19, noting that they are both safe and effective [2]. These vaccines were tested in several countries on tens of thousands of participants in clinical trials before their approval for emergency use authorization (EUA). Absent EUA, the willingness of individuals to receive a COVID-19 vaccine may depend on, among other factors, their education about and attitude toward the vaccine, as well as their insurance coverage [20]. The discovery of adverse effects caused by long-term use of COVID-19 vaccines, the emergence of new viral strains, vaccine resistance, and unpredictable epidemiological trends due to COVID-19 infection, are some of the factors that may affect an individual's willingness to receive a COVID-19 vaccine [2,12].

The safety of vaccines is continually being monitored through post-authorization safety studies, such as the ‘pandemic COVID-19 pharmacovigilance’ program, which will only end when relevant national authorities decide that they are no longer necessary [2,12]. As far back as 2003, WHO created the ‘Vaccine Safety Net’, which is a network of websites that provide reliable information on vaccine safety, available for open use by HCWs and the public [2,12].

Several regulatory agencies and governmental health bodies have been publishing freely and regularly updated COVID-19 safety reports on their websites, showing data on suspected or confirmed adverse events related to COVID-19. For example, in its ‘European Suspected Adverse Drug Reactions Database’, the EMA monitors the safety of EU-authorized COVID-19 vaccines [12]. That database collates side effects that patients and HCWs have spontaneously reported, via national medicine regulatory authorities and pharmaceutical companies, as well as any rare side effects, to the EU pharmacovigilance database ‘EudraVigilance’ [12]. In the US, the Centers for Disease Control and Prevention (CDC) established expanded safety monitoring systems’ [12] to continually monitor adverse events that might not be observed in clinical trials. Some of those adverse effects may include allergic responses, or other localized responses following vaccination, differing slightly depending on the vaccine that is administered [2].

Vaccine hesitancy can be minimized if the benefit-to-risk ratio can be argued by HCWs to patients before they receive a vaccine, to build trust and to receive boosters at a later date when needed, but this requires continuous monitoring and open reporting of actual or possible adverse effects of vaccines [2,12].

In this section, we continued to emphasize the importance of messaging, but focused on the need for public health agencies to constantly update policies and educate the public about findings and their rationale for policies, prior to their implementation.

## Mixed messaging about COVID-19 vaccine effectiveness amplifies hesitancy

In the first two years of the pandemic, one of the main concerns by vaccination-hesitant individuals was the possible long-term adverse effects of COVID-19 mRNA vaccines, given their novelty and the rapid development of COVID-19 vaccines relative to the periods required for the development of vaccines in prior pandemics [21]. Regarding the safety of mRNA-based COVID-19 vaccines, the American College of Physicians argued that given the long-term study of mRNA technology, *“mRNA vaccines will not damage recipients' genes”* [22].

However, the opinions of reputable scholars are not enough, and the public requires tangible evidence to affect a potentially life-saving decision, so robust, evidence-based clinical trial-derived data may convince some vaccination-hesitant individuals that mRNA vaccines are effective and safe [23], the latter being a strong reason for the unwillingness of these individuals to receive vaccines [23]. Allergists must thus show data-driven evidence to support the levels of negative reactions and allergic responses to different vaccines [24] and also effectively communicate that information to the public in a way that does not induce fear or panic, especially among psychologically vulnerable individuals [25]. For example, pregnant and/or lactating women might be concerned about possible negative effects of vaccines on their unborn children, or even on their bodies and/or immune systems [26], so cross-cultural studies may appease concerns.

HCWs, especially primary care providers and occupational practitioners, should be in charge of messaging to address fear and skepticism at their workplaces. In Italy, even though there was high awareness of COVID-19 vaccines among occupational physicians, their critical role in building trust toward these vaccines among workers was limited [27]. COVID-19 vaccination may be a public health measure established by central governments during a state of emergency, and may also include containment measures for unvaccinated people, but after the emergency, vaccination should become an occupational preventive measure that employers implement based on risk assessment, taking into consideration the specific modalities of exposure to SARS-CoV-2 in actual working conditions [28]. Six countries (Denmark, Israel, Italy, France, Germany and Switzerland), after introducing COVID-19 certification that required proof of vaccination, a recent negative test, or proof of recovery, showed increased levels of vaccination, especially among people younger than 30 years [29].

The second key parameter of cost–benefit analyses of vaccines that are needed to build effective public health interventions is a measure of their effectiveness. During mass vaccination campaigns, policymakers evaluated the safety-to-effectiveness ratio at the community level, and even though vaccines were found to be highly effective in preventing severe forms of SARS-CoV-2 infections in all age groups, priority was given to the ‘vulnerable’, a group that encompasses select individuals with a high risk of developing severe disease and death, including residents in long-term facilities, HCWs, the elderly, immune-depressed people, and individuals with co-morbidities [30]. Therefore, vaccination was mandatory in certain workplace settings in many countries, such as Australia, Brazil, Canada, France, Indonesia, Italy and the UK [29]. In Italy, emergency laws mandated temporary suspension from work – with the right to maintain an unpaid job – for unvaccinated teachers, police officers, military personnel, HCWs, and other specific categories of workers at a high risk of contracting and spreading COVID-19 to third parties such as patients, clients, colleagues, etc. [31].

Mandatory vaccinations in HCWs need to balance not only their benefit but also that of their patients against the risks of staff quitting and possibly increasing staff shortages [32]. Vaccine mandates are not uncommon. Before COVID-19, in many countries, a few vaccines were mandatory for children and/or for attending schools or the workplace [32]. According to a recent WHO guideline, vaccine mandates should balance their necessity and proportionality, public trust, ethical process of decision-making, safety and effectiveness, and sufficient supply of injections [33]. According to the EU health and safety laws, vaccines may be mandated after risk assessment. Occupational physicians, during medical examinations, should verify those employees that should be vaccinated, assessed by a combination of individual health conditions and the occupational risk of getting infected [34].

In this section, we argued the importance of the consistency of messaging. A disconnect between scientific findings and implemented policies, even more so if they are mandatory, may induce vaccine hesitancy, and policies that are inconsistent with actual evidence induce skepticism and mistrust.

## Implementation of mandatory policies to overcome vaccine hesitancy

There is a group of vaccine-resistant individuals who reject or resist any pharmaceutical products, including vaccines, either because they are distrustful of these products, the pharmaceutical industry, government agencies, including national health agencies, or of scientists, independent of the evidence that is presented or the effectiveness of messaging [35,36].

In a group of adults from Ireland and the UK, vaccine hesitancy was as high as 35% and 31%, respectively, with the highest skepticism caused by mistrust in scientists, followed by HCWs, in the Irish population while the UK population ranked distrust in HCWs as the primary reason [36]. Close examination of the 35 and 31% vaccine hesitancy revealed that 26 and 9% were hesitant and resistant to vaccines for the Irish population, or 25 and 6% for the UK population. Transnational data and policies might have a global effect, in which positive outcomes of clinical trials, or positive data (e.g., lack of allergic reactions in certain age groups or children) in one country might influence policies and public opinions in an unrelated part of the world.

There are ethical and legal challenges to mandating vaccines, even when they have received EUA status [37]. However, mandatory vaccinations may be socially and ethically acceptable only if they are based on a rigorous evaluation of vaccine safety and effectiveness [11]. The need to vaccinate children under 12 years old, for instance, was criticized because infection is generally mild in children and because hospitalization and life-threatening complications, such as systemic inflammatory syndrome and neurological disorders, are rare in children [38]. If documented risk factors for severe disease in children are young age and underlying comorbidities [39], then these determinants of risk should be taken into consideration by policymakers [40].

Even though short-term severe consequences of COVID-19 are much less common in children than in older adults, the risk of negative long-term COVID-19 effects (i.e., long COVID, multisystem inflammation or Kawasaki disease) is greater in children than the potential risks associated with COVID-19 vaccines [41]. Therefore, it is difficult for policymakers to make informed decisions on whether children should receive COVID-19 vaccines even though the vaccination of adolescents was suggested as a way to protect society, including older adults, by decreasing household transmission of COVID-19 infection [40]. Therefore, an evidence-based evaluation of a vaccination campaign should also be based on its impact on the community, which could be measured by evaluating several factors, such as direct effects on individuals, indirect effects (e.g., population immunity and household transmission) on unvaccinated individuals, epidemiological changes of SARS-CoV-2 and its variants, and other benefits derived from improved health [42]. In other words, public intervention, such as laws on mandatory vaccination aimed at containing the spread of the virus throughout communities would need to carefully evaluate cost-effectiveness at individual and community levels, while correcting health disparities and structural inequalities, to address the concerns of vaccine-resistant members of the public [43].

Data to assess the effectiveness of COVID-19 vaccines, and thus their mandates, can be drawn from cluster-randomized controlled or step-wedge trials, or high-quality observational data, in a less damaging way than from *“non-pharmaceutical interventions such as lockdowns”* [32]. In democratic countries, the right to individual self-determination has to be balanced with, but subordinate to, the duty to ensure public safety [44]. Therefore, to avoid the need to revert to coercion to overcome COVID-19 vaccine hesitancy [45], the obligation to get vaccinated needs to be based on reliable and shared scientific data that attests to the safety and effectiveness of a vaccine, specifically for workers and the strata of the population to which a mandate is directed.

## Data sharing: a corner-stone for COVID-19 policy-making

Data sharing during global health emergencies like the COVID-19 pandemic has been endorsed by the WHO, which called for data sharing between national stakeholders to generate reliable evidence on the safety of COVID-19 vaccines and to carry out effective and coordinated public health interventions [28,46].

In Italy, for example, the Decree-law No. 2 of 14 January 2021 regulated some information systems that are instrumental to implementing a national strategic vaccination plan for the prevention of SARS-CoV-2 infections. Indeed, the Istituto Superiore di Sanità (ISS)-Epicentro publishes reports with the combined analysis of data from the Italian National Vaccination Registry and the COVID-19 integrated surveillance system, to analyze the effectiveness of COVID-19 vaccination, i.e., the likelihood of being infected, hospitalized, admitted to an intensive care unit, or dying from SARS-CoV-2, and the persistence of vaccine-induced protection over time [47].

The WHO also invited member states to share and reuse health-related data for research purposes [28,46]. A study highlighted the risk factors associated with the mortality of patients with COVID-19 requiring treatment in intensive care units (ICUs) [48]. Another study showed that comorbidities such as vascular diseases (i.e., hypertension, diabetes mellitus, and chronic renal disease) and immunosuppression status are associated with severe COVID-19 in fully vaccinated individuals [49]. However, identifying underlying modalities that make vaccinated individuals susceptible to ICU hospitalization, such as immunization profile, type of vaccine and SARS-CoV-2 variant, age, and pattern of comorbidities, requires the collection, analysis, and sharing of data to calculate the relative risk hospitalization and ICU admission in vaccinated versus unvaccinated individuals [50].

A study comparing fully vaccinated and boosted patients with unvaccinated patients admitted to an ICU showed decreased mortality in fully vaccinated and boosted patients compared with unvaccinated patients, even though the former group was older and had a higher rate of pre-existing end-stage renal disease and a higher immunocompromised state [51]. Such studies are important to address the arguments made by anti-vaccination individuals, allowing policymakers to explain to the public why a high number of vaccinated individuals are admitted to ICUs.

In a health policy model, an attempt was made to try and understand how risk compensation may or may not affect the overall benefit of COVID-19 vaccines [52]. To achieve this, data on the efficacy of vaccination in some occupational or high-risk groups must be rapidly generated, and by including studies on immune response (and its durability and relevance for emerging variants) after vaccination, data on COVID-19 infection and deaths after vaccination by groups, and any excess mortality trends within these groups [52].

Open data sharing by public health stakeholders and researchers may facilitate and strengthen the work of official health authorities, who represent the most widely used and largely trusted source of information about COVID-19 vaccination by the public [17]. Despite these principles, even with open data, vaccine hesitancy may persist. In Canada, open data policies for COVID-19 had *“limited utility due to varying case definitions, heterogeneous and dynamic testing criteria, lack of appropriate standardization accounting for dynamics, sizes, and characteristics of the populations being tested”* [53].

Evidence-based decision-making emphasizes, using data and experiential evidence, decisions that require multiple yet balanced perspectives. The collection, analysis, and sharing of precise medically relevant data is relevant to EBM, shifts from therapy to prevention, and is centered on ‘clinician-to-patient communication’ and ‘citizen-centered healthcare’ [54].

For these reasons, data originating from scientific research, as well as scientific rigor in analyses, combined with data sharing through open and official databases developed by national and international institutions, are relevant requisites for the evidence-based justification of decisions taken by public health stakeholders.

## Evidence-based medicine for COVID-19 policy-making

EBM in public health is a recognized strategy for integrating scientific evidence into the decision-making process by policymakers [55]. The principles of EBM in public health note that decision-making should be based on the most robust available scientific evidence, by balancing the impact of interventions, including vaccines, with the benefits to the community [55]. Some authors claimed that EBM for COVID-19 is still limited and controversial [56] and balancing socio–economic disruption with psychological and physical consequences to the health of individuals, through precise cost-effectiveness analyses, is still challenging [57]. Balancing the benefits and harms of COVID-19 vaccines requires high-quality research [57]. Thus, EBM-based systematic reviews and meta-analyses are needed to synthesize the existing scholarly literature related to the ongoing pandemic [58]. Systematic reviews may struggle to capture the real-world context of public health interventions in the case of complex diseases [59]. Moreover, linking clinical effectiveness to cost effectiveness at the population level is challenging because randomized clinical trials are not always applicable for investigating public health concerns [58]. Therefore, COVID-19 technology-enabled living systematic reviews are needed to enhance knowledge translation in clinical practice [59].

## Importance of the EBM-open data link

One robust solution to address misinformation-based vaccine hesitancy is to focus on strengthening communication between institutions and the general public using EBM, which combines a critical appraisal of scientific evidence with patients' preferences through shared decision-making [60]. More importantly, an upgrade to a ‘platinum’ model of open data policies can mitigate scientific uncertainty by eliminating unreliable sources of scientific knowledge [61], as occurred in leading medical journals, the *Lancet* and *New England Journal of Medicine*, which published papers that included unreliable data sourced from Surgisphere that had not been properly vetted by those journals [62]. A freely accessed open and public data-based international platform coordinated by WHO, where constantly updated information on vaccines' adverse effects is freely reported alongside updated information on vaccines' effectiveness (e.g., with the frequency and severity of infection in vaccinated people by age, gender, and co-morbidities) would allow scholars to draw relevant data for targeted cost-effectiveness analyses and perhaps overcome fear and vaccine hesitancy [63]. Providing up-to-date information to the public and health providers about rigorous measures taken before the introduction of new vaccines, and open data by post-marketing surveillance of vaccine-related events, may counter vaccine hesitancy [64]. Indeed, open data observations on the safety and effectiveness of vaccines, provided with transparency through tailored communication strategies, may increase the uptake of COVID-19 vaccines among HCWs [65], who are generally the most trusted advisors regarding vaccination-related decisions for a population [66]. After a state of emergency, EBM-based open data related to the safety and efficacy of COVID-19 vaccines is necessary before they are mandated.

Pooled publicly available datasets are invaluable and necessary, even if there are privacy-related issues with patient-level COVID-19 data, an issue that can be dealt with relatively easily by blinding data [67]. Sharing open data on vaccines has some disadvantages due to the potential misuse of data because anyone can provide interpretations of that data, with the potential of increasing confusion or conveying conflicting messages to the public, while social media can boost incorrect or misleading interpretations of scientific findings [67]. Yet, open data is needed to monitor the safety of vaccines internationally [17] and mitigation measures should include open data from institutional and well-referenced scientific platforms to avoid this pitfall.

The lack of transparency can undermine trust in science and public health institutions by the public, and increase conflicts between governments and their citizens, so maintaining the public's confidence in public health leaders and authorities is essential for an effective response to COVID-19 [68]. Decisions should be made openly and transparently, data should be accessible for scrutiny and auditing, and the public should be able to access this information through appropriate communication channels [69]. The public could be updated with scientific information, but this requires work to increase access to digital technology such as artificial intelligence [70]. For instance, by calculating vaccination rates and stratifying by age groups in a population, it may be possible to forecast vaccine effectiveness versus severe disease, thus improving the responsiveness of a healthcare system [71]. Robust data are needed for epidemiological analyses and mathematical models, which are useful tools to make predictions, provided that they are evidence-based [72], but a global health response requires scientific and political collaboration among countries and the open exchange of information. Absent robust open data models, retracted COVID-19 papers, such as the Surgisphere-related papers, may continue to be cited, lending credence to statements based on unreliable data [73] and fueling the vaccination-hesitant sector of society.

Ultimately, open data sharing may generate EBM information that may allow policymakers to take evidence-based decisions and establish health-based national policies for vaccines, or policies that suppress or mitigate COVID-19, avoid ethical transgressions caused by rapid publication coupled with low-quality or superficial peer review, representing a threat to the integrity, accuracy, and value of the scientific literature [74], and to ultimately gain the public's trust [17]. Open data that is freely and efficiently provided by policymakers, similar to strategies suggested for journals and their editors [75], is an essential value to guide ethics-based decision-making in this and future pandemic crises. Risk to integrity could be tackled, to some extent, by mandatory declarations of conflicts of interest by authors, reviewers, and editors, especially if they have links to pharmaceutical companies [63,76], as well as fortified and careful verification of data and facts during every step of peer review and paper publication-related processes [75].

In the era of electronic health records, inferences drawn from open research and shared data [67], as encouraged by the WHO [77], provide a useful and robust infrastructure that may inform epidemiological inquiries and guide treatment protocols when clinical trial data does not exist. This is a valuable contribution to addressing vaccine hesitancy in a population and promoting high vaccination rates during the ongoing global mass vaccination campaign against new and old COVID-19 variants. Evidence-based and data-driven information may address online vaccine misinformation, which is present in news outlets, websites, and social media, and is often disseminated by artificial intelligence [78]. Vaccine hesitancy was higher among individuals that selected unmonitored media platforms than individuals that relied on information from ‘source-verified media platforms’ [79].

At the start of the pandemic, the G7 Science and Technology Ministers released a declaration calling on governments to provide their researchers with *“timely access to advanced experimental and computing resources and artificial intelligence tools”* as well as *“effective and safe COVID-19 diagnostics, therapeutics, healthcare interventions, vaccines, and personal protective equipment”* [80].

The EU's COVID-19 Data Portal [81], which is a COVID-19 open data repository, can be used by researchers, data scientists, organizations, and policymakers [82]. This portal was created to facilitate open data sharing of COVID-19 research providing updates on drug targets, vaccines, and effects of new variants, to facilitate policymakers to design better public health responses, according to FAIR (findable, accessible, interoperable, and reusable) [82], TRUST (transparency, responsibility, user focus, and technology), and CARE (collective benefit, authority to control, responsibility, and ethics) principles of open science [83,84].

## COVID-19 ‘noise’, misinformation & disinformation

The COVID-19 infodemic is often characterized by an ‘information overload’, which, in the context of the COVID-19 pandemic, poses challenges not previously encountered [85,86]. Infodemiology is recognized by public health organizations and the WHO as an important ‘emerging scientific field’ and ‘critical area of practice’ during a pandemic [87]. In this state, false news, conspiracy theories, claims of magical cures, and racist views can be widely shared, including via social media, with the potential to increase anxiety and stress [86]. Some social media platforms provide users with the ability to report inappropriate content [88]. As an example, a popular conspiracy theory linked 5G to the spread of COVID-19, leading to the burning of 5G towers in the UK [89].

‘Noise’, i.e., irrelevant or uninformative information, in an ‘infodemic’ risks degrading the effectiveness of communication of public health policies that are put in place by policymakers [85]. Contradictory information spread by virologists and medical experts, and talk shows where confounding messages are conveyed by celebrities, as well as claims not backed by evidence but spread through social media by anti-vaccination proponents may be cited in scientific literature and affect public behavior and attitudes, a phenomenon that already existed prior to the COVID-19 pandemic, undermining efforts made by policymakers and people's trust in governments' strategies [90]. In contrast, ‘trusted messengers’ who can influence vaccine-hesitant individuals, should effectively disseminate the risks and benefits of vaccinations while positive opinions of family, friends, and health professionals may be associated with a higher willingness to get vaccinated [22].

Furthermore, ongoing changes to rules and public health regulations in a pandemic, needed to protect public health, are established based on ever-changing epidemiological variations and may create confusion and mistrust by the public in policymakers, as they see their everyday lives practically affected. In Austria, vaccine hesitancy was linked to mutual distrust in vaccines and authorities [91]. In times of uncertainty and anxiety, conspiracy theories about the existence of the SARS-CoV-2 and the effectiveness of vaccines to tackle this pandemic works for a sector of the public because they receive ‘comfort’ from an explanation [85], even more so in people with low health literacy [92]. HCWs play a fundamental role in tackling COVID-19 vaccine hesitancy, and the lack of action by HCWs in providing pro-vaccine and scientific information about vaccines on social media may also constitute a public health threat [93]. Vaccine hesitancy and refusal are also fueled by COVID-19 misinformation and disinformation on social media platforms [94].

Potential solutions include a strong response by governments with criminal punishment against people who create and share falsified information [92], banning them from social media platforms, and a stronger presence of national health agencies in social media, as occurred in the UK [95], the use of artificial intelligence to automatically detect fake COVID-19 news [96], leveraging eHealth literacy skills, and more specifically, media literacy [97], strategic multilateral cooperation coordinated by the USA and EU countries [98], imposing legal and ethical imperatives on social media companies [94], and developing interventional educational campaigns targeted toward populations at risk of vaccine hesitancy [99]. The infosphere is a social determinant of health, and the development of ‘infodemiologic’ skills, as well as the deployment of tailored interventions to manage the infodemic and combat disinformation, will serve the public well and protect it from disinformation [100]. Open and effective health communication is likely to be the best strategy to address COVID-19 vaccine hesitancy and this should be based on open data sharing and the right to communicate about vaccines' safety and effectiveness [101]. However, data sharing will only improve evidence-based policies when it is mandated.

There are three main tools to contain the COVID-19-related burden on healthcare systems: High rates of vaccination in a population, lockdown and mitigation measures, and a population's compliance with these mitigation measures through safe individual behavior. This information should be based on official open data, which should be published by reliable and institutional sources [102]. On the other hand, open data may be misused by unscrupulous scientists [74] or anti-vaccination movements that may undermine the achievements of scientists and policymakers [90]. Open data that is freely and efficiently provided by policymakers, similar to data-related strategies suggested for journals and their editors [17], is an essential value to guide ethics and evidence-based decision-making, as the basis of national and global health policy strategies, in this and future pandemic crises. Therefore, mandated data sharing is not only timely but also necessary.

## Conclusion

Mixed messaging about COVID-19 vaccine effectiveness and misinformation amplify COVID-19 vaccine hesitancy. Open data sharing and evidence-based policies established by policymakers, including about boosters, may address COVID-19 vaccine hesitancy and increase vaccine coverage worldwide. Mandatory vaccinations may be socially and ethically acceptable only if they are based on a rigorous evaluation of vaccine safety and effectiveness.

## Future perspective

In forthcoming years, policymakers and stakeholders can address vaccine hesitancy by tackling public misinformation and by building public trust. An evidence-based policy approach in public health that uses a ‘platinum’ model based on global open data sharing between scientists and policymakers is both timely and necessary.

**Executive summary**

**Sources of COVID-19 vaccine hesitancy & the importance of effective messaging**

- False beliefs or attitudes about health and prevention, and low levels of knowledge and awareness of recipients are sources of vaccine hesitancy.
- Inadequate communication by public health stakeholders lowers vaccine acceptance and adds to vaccine hesitancy.
- Effective messaging by public health stakeholders is important to tackle COVID-19 vaccine hesitancy.

**Constantly updated policies & public education to combat vaccine hesitancy**

- Communication, culture, politics, and barriers to accessibility, as well as individual and group differences, impact vaccine acceptance.
- Irrelevant or uninformative information dampens the ability to effectively communicate public health policies that are put in place by policymakers during the COVID-19 pandemic.
- Updating policies and improving public education is needed to combat vaccine hesitancy.

**Mixed messaging about COVID-19 vaccine effectiveness amplifies hesitancy**

- Prior to vaccines, concerns were spread, but were unfounded.
- During vaccine development and during global vaccination campaigns, sometimes contradictory messages were provided to the public.
- Mixed messaging about COVID-19 vaccine effectiveness amplifies hesitancy.

**Implementation of mandatory policies to overcome vaccine hesitancy**

- Where vaccine hesitancy is high, a more stringent approach is needed to ensure uptake.
- In such cases, mandatory vaccines may be necessary.
- Risks of claims of suppressed freedoms may arise.

**Data sharing: a corner-stone for COVID-19 policy-making**

- Mandatory policies to overcome vaccine hesitancy should be based on open data sharing.
- This requires careful coordination by public health stakeholders.

**Evidence-based medicine for COVID-19 policy-making**

- An evidence-based open dataset of policies is needed to try and reduce vaccine hesitancy.

**Importance of the EBM-open data link**

- EBM-based policies that use open data would allow policymakers to take evidence-based decisions and establish politics-free, health-based national policies regarding vaccines.
- Preprints are a useful and quite open access and open data way to share findings rapidly, but more stringent measures to vet them to avoid the amplification of false signals related to misleading policies or false findings are needed.

**COVID-19 ‘noise’, misinformation & disinformation**

- An overwhelming amount of information may cause an ‘infodemic’, and irrelevant or uninformative information may also confuse the public.
- Related to publishing, it is essential for authors to declare potential conflicts of interests.
- Editors play an important role in blunting ethical transgressions by stricter data validation while taking the balance between the speed of publication and benefits to society's health into account.
